# Antifungal Drug-Drug Interactions with Commonly Used Pharmaceutics in European Pediatric Patients with Acute Lymphoblastic Leukemia

**DOI:** 10.3390/jcm12144637

**Published:** 2023-07-12

**Authors:** Beata Sienkiewicz-Oleszkiewicz, Małgorzata Salamonowicz-Bodzioch, Justyna Słonka, Krzysztof Kałwak

**Affiliations:** 1 Department of Clinical Pharmacology, Faculty of Pharmacy, Wrocław Medical University, ul. Borowska 211a, 50-556 Wrocław, Poland; 2 Department and Clinic of Pediatric Oncology, Hematology and Bone Marrow Transplantation, Wrocław Medical University, Borowska 213, 50-556 Wrocław, Poland; malgorzata.salamonowicz@umw.edu.pl (M.S.-B.); krzysztof.kalwak@umw.edu.pl (K.K.); 3 Gilead Sciences Poland Sp. z o.o., ul. Postepu 17A, 02-676 Warsaw, Poland; justyna.slonka@gilead.com

**Keywords:** acute lymphoblastic leukemia, antifungal agents, children, drug interactions, liposomal amphotericin B

## Abstract

Leukemia is one of the leading childhood malignancies, with acute lymphoblastic leukemia (ALL) being the most common type. Invasive fungal disease is a concerning problem also at pediatric hemato-oncology units. Available guidelines underline the need for antifungal prophylaxis and give recommendations for proper treatment in various clinical scenarios. Nonetheless, antifungal agents are often involved in drug-drug interaction (DDI) occurrence. The prediction of those interactions in the pediatric population is complicated because of the physiological differences in adults, and the lack of pharmacological data. In this review, we discuss the potential DDIs between antifungal agents and commonly used pharmaceutics in pediatric hemato-oncology settings, with special emphasis on the use of liposomal amphotericin B and ALL treatment. We obtained information from Micromedex^®^ and Drugs.com^®^ interaction checking databases and checked the EudraVigilance^®^ database to source the frequency of severe adverse drug reactions that resulted from antifungal drug interactions. Several major DDIs were identified, showing a favorable safety profile of echinocandins and liposomal amphotericin B. Interestingly, although there are numerous available drug interaction checking tools facilitating the identification of potential serious DDIs, it is important to use more than one tool, as the presented searching results may differ between particular checking programs.

## 1. Introduction

Leukemia is the most common childhood malignancy worldwide [1]. It accounts for 27% of childhood cancers in the United States, 35% in China and 30–33% in Europe [2,3,4,5,6]. The incidences for various leukemias are the following: acute lymphoblastic leukemia (ALL), between 43.2 and 44.9 per million; acute myeloid leukemia (AML), between 9.8 and 10.6 per million [7,8]; chronic myeloid leukemia (CML), 2.5 per million; and unspecified leukemia (UL), 0.5 per million [7]. Acute lymphoblastic leukemia is the most common type. The peak incidence is between 2 and 5 years of age. The exact cause of ALL is not known. The survival rate of pediatric ALL is very good and it has improved to approximately 90% in recent years due to trials based on risk stratification by biologic features of blasts and response to therapy, treatment modification based on patient pharmacodynamics and improving supportive care [9].

In the United States, there were 160 regimens for ALL evaluated during the last fifty years in phase 3 trials conducted by the Children’s Cancer Group and the Pediatric Oncology Group. Similarly, there are many trials ongoing in Europe conducted by the following cooperative pediatric groups: International Berlin–Frankfurt–Münster Study Group (I-BFM-SG), United Kingdom Acute Lymphoblastic Leukemia (UKALL), French Acute Lymphoblastic Leukemia Study Group (FRALLE), and Italian Association of Pediatric Hematology and Oncology (AEIOP) [10,11,12,13,14]. Despite some differences between protocols, most are based on a similar schema of drugs including corticosteroids, daunorubicin, doxorubicin, vincristine, L-asparaginase preparations, methotrexate, cyclophosphamide, mercaptopurine and cytarabine. New drugs include bortezomib, ruxolitinib, nelarabine, blinatumomab, inotuzumab ozogamicin and tisagenlecleucel. Despite promising results presented by the mentioned research groups, there is still a number of children who die due to treatment toxicity. In addition to individual predisposition, drug-drug interactions (DDI) are of great importance for understanding complications. Knowledge of these can significantly improve long-term prognosis by reducing adverse effects [12,14,15,16].

Invasive fungal disease (IFD) is common in hemato-oncology settings. A study published in 2003 showed that it is responsible for one fifth of the infectious deaths in children with ALL [17]. The incidence of IFD is between 8 and 9.7%, and increases significantly (reaching 23.5%) in patients with relapsed/refractory ALL and treated with high-risk (HR) compared to standard-risk ALL chemotherapy protocols [18,19]. IFD occurrence was shown to be connected with a delay of treatment, or significant reduction in dosing and overall longer hospital stay [18,20]. This is why antifungal agents are used, for the prevention and treatment of IFD, concomitantly to ALL treatment protocols.

Unfortunately, antifungal drugs are frequently involved in DDIs with leukemia treatment, so there is also an ongoing need for drug-drug interaction identification and management. Klein et al. evaluated all available infection prophylaxis guidelines for pediatric AML patients within the I-BFM-SG. Despite a long history of close collaboration in this large international consortium, many differences were observed between the affiliated groups. Those were mainly connected with bacterial prophylaxis and variations in pharmacological antifungal prophylaxis. As a result, a distinct distribution of DDIs among hospitals may occur. Therefore, it is important to characterize potential DDIs, especially in the pediatric population, to ensure safe and effective drug use [21].

Currently, the following antifungal drugs are recommended for IFD prophylaxis: fluconazole (FCZ), posaconazole (PCZ), itraconazole (ICZ), myconafine (MYC), and liposomal amphotericin B (L-AmB), However, L-AmB is not approved for prophylactic purposes. According to the 8th European Conference on Infections in Leukaemia (ECIL-8) guidelines, IFD prophylaxis should be conducted in patients with high risk of invasive fungal disease (incidence of IFD > 10%) and in patients with lower risk, after positive individual assessment. For treatment purposes, ECIL-8 guidelines recommend polyenes, echinocandins and triazole antifungal drugs, depending on clinical symptoms and detected pathogens. L-AmB and caspofungin (CASP) are recommended in empirical and preemptive therapy. MYC, anidulafungin (ANID), VCZ, and FCZ may be used in the treatment of invasive candidiasis (IC) and VCZ, L-AmB, and isavuconazole (ISZ) in the treatment of invasive aspergillosis (IA). L-AmB and ISZ are recommended for the treatment of mucormycosis. In this type of infection, ISZ has only a provisional recommendation, as it is not approved in the pediatric population. Lower grade recommendations for the treatment of IC, IA and mucormycosis were given for amphotericin B lipid complex (ABLC), ABLC and VCZ combined with ANID and L-AmB combined with CASP or PCZ, respectively [22].

Other guidelines, like Clinical Practice Guideline for Systemic Antifungal Prophylaxis in Pediatric Patients With Cancer and Hematopoietic Stem-Cell Transplantation Recipients recommend an echinocandin, voriconazole, or itraconazole for children younger than 13 years of age, and posaconazole as an additional option in children 13 years of age and older [23].

For antifungal DDI identification, special tools were developed in the last few years [24]. However, proper antifungal management in the pediatric population may be challenging because of differences in the pharmacokinetic and pharmacodynamic properties of drugs. For example, the activity of the CYP2C19 isoenzyme responsible for voriconazole metabolism changes during childhood. CYP2C19 has a reduced activity between birth and 6 months of age. Afterwards, it reaches the adult activity in early infancy, before exceeding the adult levels during childhood and returning to those in adolescence [25]. Changes in enzymatic activity may impact the dosing of susceptible drugs, but will also influence potential drug-drug interaction (PDDI) occurrence. Another challenge connected with the use of interaction checking databases is the alert fatigue phenomenon, leading the prescriber to habitual overriding of alerts due to detection of multiple interactions that are sometimes not relevant from a clinical point of view [26].

Also in many cases, due to lack of data obtained from the pediatric population, the DDI information is extrapolated from the adults. As a result, erroneous conclusions may be drawn due to differences between these two populations [27].

This assumption is supported by the results of a study assessing the prevalence and exposure profile of potential drug interactions in infants, children and adolescents during hospitalization. Of 498,956 hospitalizations in 2011, 49% were associated with at least one PDDI. The majority of DDIs was life-threatening or required medical intervention. Seventeen percent of PDDIs were connected with the use of anti-infective drugs, including antifungal agents [28].

In another retrospective cohort study evaluating polypharmacy and PDDI among pediatric patients in the intensive care units in the United States, 51.1% of identified PDDI were considered as major, and 18.1% were connected with the use of anti-infective agents [29].

The prevalence of DDIs in hospitalized pediatric hemato-oncology patients ranges from 44.7–51.3% to 56.8–74.1, depending on the database, and may reach 83.5% in outpatients. Regardless of the in- or outpatient status, antifungal drugs are frequently involved in DDI occurrence, especially in this patient group [30,31,32].

Another problem associated with managing pediatric blood cancer patients is the frequent need for off-label use of drugs. This hassle also appears during antifungal treatment and may result in unintended DDIs [33,34].

This article aims to evaluate the DDIs between antifungal agents and commonly used pharmaceutics in European pediatric patients with acute lymphoblastic leukemia. Special attention is paid to discrepancies between interaction checking programs and indication of drugs having the lowest numbers of drug-drug interactions.

## 2. Materials and Methods

To identify potential DDIs, we obtained information from 2 large interaction checking databases, Micromedex^®^ https://www.micromedexsolutions.com (accessed on 7 October 2022) and Drugs.com^®^ Interaction Checker https://www.drugs.com/drug_interactions.html (accessed on 7 October 2022). We deliberately chose one database that is commercial and one non-commercial. The non-commercial Drugs.com^®^ Interaction Checker is often used due to its accessibility. Micromedex^®^, on the other hand, is a commercial, frequently used interaction checking program, with a good ability to detect clinically important DDIs [35].

The list of drugs used in pediatric ALL treatment was determined by reviewing Polish therapeutic protocols and those of other European countries.

In addition, we also checked the EudraVigilance^®^ https://www.adrreports.eu/ (accessed on 13 February 2023) database to source the frequency of major adverse drug reactions spontaneously reported in this European database, caused by antifungal drug-drug interactions in children. EudraVigilance^®^ is a source of information on managing and analyzing suspected adverse reactions to medicines on the basis of spontaneous adverse reaction reports obtained from healthcare professionals, marketing authorization holders and patients. It allows, e.g., to search for adverse reactions regarding their seriousness, and population characteristics like age, gender, kind of reported suspected reaction and date of the report. However, it does not give information on the primary health condition of the patient, unless stated in the report form.

## 3. Results

Table 1 shows potential, clinically relevant drug-drug interactions between antifungal drugs and acute lymphoblastic leukemia treatment, together with their classification in the searched drug interaction checking databases.

Table 2 presents the DDI’s share in the total number of serious ADR notifications in the pediatric population from 2017 to 2022 based on EudraVigilance^®^ reports.

### 3.1. Dissimilarities in DDI Detection and Classification between Databases

Analyzing Table 1, we noticed that in some cases, one database detects an interaction and the other one does not. The Micromedex^®^ database, for example, detects a major interaction between ifosfamide fluconazole (FCZ), posaconazole, and voriconazole. The Drugs.com^®^ Interaction Checker database gives no information about a possible interaction. According to the product information (PI) of ifosfamide, the concomitant use with drugs inhibiting the CYP3A4 isoenzyme, may result in the alteration of the effectiveness and toxicity of ifosfamide. This is primarily connected with greater central nervous system toxicities and nephrotoxicity. FCZ, PCZ and VCZ are moderate up to potent inhibitors of CYP3A4, what supports a major DDI classification [24,36].

In several cases, there is an inconsistency between the DDI classification in the included databases. This is especially important for interactions marked as major or eventually contraindicated (depending on the checking program). Of note, both databases use different definitions describing the severities of interactions. For example a DDI marked as major in the Drugs.com^®^ Interaction Checker database does not mean the same as major interaction identified by the Micromedex^®^ database. The definitions of severity in both databases are presented in the footnotes of Table 1.

A clinically relevant example of such a difference is the Micormedex^®^ database identifying a major interaction between vincristine and all triazole antifungal agents except of isavuconazole (ISZ). At the same time, the Drugs.com^®^ Interaction Checker database identifies a moderate risk of interaction for fluconazole and isavuconazole, and a major risk for all other triazole antifungal agents. In this case, the risk evaluation is particularly important because DDIs of azoles with vincristine may lead to severe adverse drug reactions.

In an analysis of reported adverse drug interactions (ADIs) between vincristine and azoles by Moriyama et al., the median age of patients presenting with DDIs was 8 years, and the majority (70%) was diagnosed with ALL. The review showed that the onset of ADIs most rapidly occurred after itraconazole (9.5 days), followed by posaconazole (13.5 days) and voriconazole (30 days). ADIs resulted in peripheral and autonomic neuropathy (59.6% and 38.3%, respectively), hypertension (29.8%), gastrointestinal side effects (66%), electrolyte abnormalities, hyponatremia, syndrome of inappropriate antidiuretic hormone secretion (SIADH) (44.7%) and seizures (23.4%), depending on the drug used [37]. Although those ADIs caused by DDIs are known by experienced clinicians, it is important to emphasize them, as they are not particularly described in the ALL treatment protocols. Some antifungal drugs, like posaconazole oral suspension, are used off-label in most children with ALL due to their approval regarding age [34]. This makes it difficult to predict the occurrence and the magnitude of DDIs, as safety data from large randomized multicenter studies are usually missing. Two studies, one performed among pediatric and one among adults with ALL, demonstrated an increase in side effects of vincristine in patients receiving fluconazole. Unfortunately, the pediatric study failed to perform a comparison of the severity of vincristine toxicity caused by different azoles including FCZ due to small numbers of patients [38]. In the adult population analysis, decreased peristalsis symptoms occurred in 50% of patients treated with vincristine and fluconazole. Also, in this study, the patient numbers were relatively small [39].

According to the product information, the impact of ISZ on the toxicity of vinca alkaloids was not studied. However, a possibility of increased toxicity exists, as the drug is a P-glycoprotein (P-gp) inhibitor, and vinca alkaloids are substrates for this protein [40].

### 3.2. Relevant DDIs of New Drugs

It is important to analyze the DDIs of new drugs, as there is usually limited data on this topic. The Drugs.com^®^ Interaction Checker database identifies a moderate interaction between imatinib and all triazoles. This interaction is primarily mediated by the inhibition of CYP3A4 isoenzyme, and may result in elevation of imatinib concentrations. In the case of concomitant voriconazole and imatinib use, there is also a possibility of an additional interaction, resulting from competitive inhibition of CYP2C9 by imatinib. Of note, VCZ is both a substrate and an inhibitor of this isoenzyme [41]. The Drugs.com^®^ Interaction Checker database also identifies a moderate interaction between bortezomib and voriconazole, posaconazole and itraconazole. This information stands in line with the PI, where attention is recommended when bortezomib is used together with potent CYP3A4 inhibitors due to the possibility of area under the curve (AUC) increase [42]. However, it should be kept in mind that vinca alkaloids are also used during bortezomib phase, so triazoles should be generally avoided due to previously mentioned major interactions with vincristine [39].

A major interaction is highlighted by both databases between ruxolitinib and all triazoles, except isavuconazole. This interaction is also connected with CYP3A4 inhibition and, in the case of fluconazole, with additional CYP2C9 inhibition. As a result, an elevation in ruxolitynib concentrations may occur [43]. It is worth mentioning that this drug is very rarely used in ALL, but can be crucial in patients with a JAK-STAT pathway mutation [15,44].

Similarly, an interaction is identified by both the checking programs between inotuzumab ozogamicin and all triazole antifungal agents with the exception of ISZ and ICZ. For itraconazole, only the Micromedex^®^ database identifies a major interaction. According to the PI of inotuzumab ozogamicin, prolongation of the QT interval may occur during drug use [45]. Triazole antifungal agents, especially VCZ, FCZ, PCZ, and ICZ were also connected with prolongation of the QT interval. Less data are available on ISZ [46,47]. Nonetheless, in each clinical scenario there is a recommendation to monitor electrolyte levels and electrocardiogram results before starting inotuzumab ozogamicin and periodically throughout treatment. Special attention should be paid to patients receiving the drug concomitantly with triazoles, as there is an increased risk for QT interval prolongation [45,48]. According to a study by Hibma et al. where QTc concentration modeling was used, drug doses of 1.8 mg m^−2^ per cycle do not pose a significant risk for clinically significant QT interval prolongation [49]. Newer studies focus on hepatotoxicity and sinusoidal obstruction syndrome induced by inotuzumab ozogamicin. As safety data on its use in children are incomplete, it is worth remembering that antifungal drugs may also cause hepatotoxicity. The use of more than one drug with a hepatotoxic component increases the risk [50].

For the new triazole antifungal agent isavuconazole, several potential DDIs are detected. The Drugs.com^®^ Interaction Checker database identifies a risk of interaction with corticosteroids, antineoplastic drugs (doxorubicin, daunorubicine, cyclophosphamide, ifosfamid, and imatinib), erythromycin, ondansetron, granisetron, metoclopramide and opioids (morphine, tramadol, oxycodone, fentanyl, buprenorphine, and methadone). ISZ is a P-gp inhibitor, so morphine, a P-gp substrate, should be carefully used in patients treated with this antifungal drug [51,52]. Furthermore, ISZ is an inhibitor of the Breast Cancer Resistance Protein (BCRP), involved in the transport of doxorubicin, daunorubicine, and imatinib. Isavuconazole is also an inhibitor of CYP3A4/5 isoenzyme responsible for the biotransformation of vincristine, erythromycin, ondansetron, granisetron, oxycodone, fentanyl and buprenorphine raising the possibility of DDIs. ISZ induces the activity of CYP2B6, potentially leading to decreased concentrations of cyclophosphamide, ifosfamide, metoclopramide and methadone [40,53,54]. Although dose adjustments of short-acting opioids and methadone are not required, according to the PI, careful patient monitoring during concomitant use is particularly important [40].

### 3.3. Other Clinically Relevant Interactions

From a clinical point of view, DDIs connected with analgesic treatment are of interest. Triazole antifungal agents are commonly involved in those interactions, whereas other antifungals (L-AmB and echinocandins) are not. The Drugs.com^®^ Interaction Checker database identifies a moderate risk of interaction between buprenorphine, fluconazole and posaconazole. Micromedex^®^ database, on the other hand, classifies both combinations as contraindicated. Buprenorphine is metabolized by the CYP3A4 isoenzyme. Caution should be certainly paid when using it concomitantly with drugs being inhibitors of CYP3A4, like fluconazole and posaconazole. In addition, all three drugs may prolong the QT interval. However, in the case of posaconazole, the risk is conditional [55,56,57].

In a study analyzing in vitro metabolism inhibition of buprenorphine, methadone and oxycodone by azole antifungal drugs, voriconazole had the greatest potential to inhibit the CYP3A4 pathway [58]. The in vitro results are reflected in a randomized, placebo-controlled crossover study aiming to determine the impact of voriconazole and posaconazole on sublingual buprenorphine pharmacokinetics. Voriconazole led to increase in pharmacokinetic values (AUC, Cmax, and t_1/2_) to a greater extent than posaconazole. The authors suggested careful patient monitoring, especially when sublingual buprenorphine is used together with voriconazole [59].

A very interesting DDI is detected only by the Micromedex^®^ database between cotrimoxazole and fluconazole, leading to QT prolongation. At the same time, according to the product information and literature search, the concomitant use of both drugs may decrease the concentrations of the biologically active metabolite of sulfamethoxazole, leading to lower numbers of adverse drug reactions, as this metabolite is connected with their appearance [57,60,61,62].

### 3.4. Issues Related to DDIs between Antifungal Drugs and All Treatment in the Pediatric Population

Lowest numbers of interactions are connected with the use of micafungin, caspofungin and liposomal amphotericin B. In Table 2, where EudraVigilance^®^ ADR reports regarding the pediatric population (0–17 years) were analyzed in a timespan from 2017 to 2022, the average percentage of serious ADIs of micafungin and caspofungin was 0.8% and 7.9%, respectively. Amphotericin B in different formulations led to overall 1.6% of ADIs (liposomal amphotericin B alone to 0.6%). Triazole antifungal agents presented the highest incidences of reported serious ADR caused by DDIs. Those were 9.8%, 11.2%, 15.4%, 18.6%, and 4.3% for fluconazole, voriconazole, posaconazole, itraconazole and isovuconazole, respectively. The relatively low number of spontaneous reports of antifungal drugs related ADIs may be connected with the already widely noticed problem of underreporting of ADRs. However, this topic is beyond the scope of the article [63,64,65]. Nonetheless, the presented values clearly indicate a need for special caution enrollment, when co-administering drugs with triazoles, especially in children.

Echinocandins are relatively safe, especially when it comes to potential DDIs. However, concerns of liver and cardiac toxicity were reported [66]. In the EudraVigilance^®^ database, two cases of QT interval prolongation were reported between 2017 and 2022 in children under micafungin treatment and zero for children treated with caspofungin. In the case of CASP, potential interactions may also appear on the OATP-1B1 transporter background [67]. Echinocandins are mainly used in treatment of *Candida* spp. infections and as second-line treatment for invasive aspergillosis. In the treatment of pediatric fungal infections, caspofungin and micafungin are approved [66]. Caspofungin and micafungin are highly protein bound, but achieve low cerebrospinal fluid concentrations, which limits their use [68]. According to the PI of caspofungin, activity against other fungal strains, apart from *Candida* spp. and *Aspergillus* spp., was not observed [69]. The use of micafungin is limited because of the potential risk of liver cancer development, first observed in rats. This potential threat is subject of a Blackbox warning found in the product’s information. However, in a recently published multicenter cohort study, with a 12 year follow-up, evaluating 40,110 patients receiving micafungin or other parenteral antifungals, the mortality rates resulting from hepatocellular carcinoma were similar between groups receiving different antifungal treatment (<0.2 per 1000 person years). In conclusion, the potential risk for liver cancer development should not play an important role in clinical decision making [70]. Overall, adverse drug reactions were observed more frequently in the pediatric population than in the adults under micafungin treatment. For caspofungin, those frequencies were similar [69]. In summary, although echinocandins have the lowest numbers of potential DDIs, their use is limited mainly by the relatively narrow spectrum of activity, lack of CNS penetration and eventual toxicity concerns [24,66,68,69,71,72]. This is particularly important in the pediatric blood cancer population.

Liposomal amphotericin B also presents a relatively limited number of potential DDIs. According to Table 1, only the Drugs.com^®^ database informs about one major interaction with foscarnet and several moderate interactions with steroids, cyclophosphamide, ifosfamide, ceftazidime, aminoglycosides, vancomycin, colistin, valganciclovir and proton pump inhibitors. Most of those moderate interactions are connected with deepening of hypokalemia and the possibility of nephrotoxicity. However, the case of deepening of hypokalemia may also favor the occurrence of QT interval prolongation. According to the EudraVigilance ^®^ database, there were four cases in children in the timespan between 2017 and 2022. For concomitant PPI use, deepening of hypokalemia is mediated through impaired parathyroid hormone secretion caused by hypomagnesemia resulting from long-term (over one year) PPI use [73]. As reported by Kyriakidis et al., liver function abnormalities may occur during liposomal amphotericin B use. However, they are usually mild, and do not make it necessary to discontinue the drug [74]. L-AmB is approved for the treatment of severe systemic and/or deep mycoses including disseminated candidiasis, aspergillosis, mucormycosis, chronic mycetoma, cryptococcal meningitis and visceral leishmaniasis. Another drug indication is the empirical treatment of presumed fungal infections in febrile neutropenic patients, where the fever has failed to respond to broad spectrum antibiotics and appropriate investigations have failed to define a bacterial or viral cause [75]. In addition, several central nervous system infections were successfully treated with L-AmB [72]. Although the drug is approved for treatment of IFD in children, there were several investigations showing its successful off-label use in prophylaxis. In a non-comparative cohort study performed among 51 pediatric patients, the drug was well tolerated and provided adequate protection against fungal infections [76]. Also, reduced frequency dosing schemes were investigated in the pediatric population, showing that dosing once per week provides effective and safe protection against IFI in children [77]. However, it is important to state that L-AmB is not approved for prophylactic purposes in children. In summary, the liposomal amphotericin B formulation has a favorable profile of potential DDIs, a broad spectrum of antifungal activity with a fungicidal mode of action, and is well tolerated in the pediatric population, especially in comparison with other amphotericin B formulations [78]. A limiting factor of the use of L-AmB is certainly the lack of approval in prophylaxis in children.

The highest potential in generating DDIs can be observed among triazole antifungal agents, being potent cytochrome P450 inhibitors. Clinically, DDIs at the stage of metabolism are very common [79]. It is estimated that 70% of them are caused by enzyme inhibition [80]. For triazole antifungal agents, especially CYP2C9, CYP2C19 and CYP3A4, are inhibited [24]. Those three isoenzymes are responsible for the metabolism of a wide variety of currently used drugs, which paves the way for numerous interactions [53]. In addition, posaconazole is also a P-glycoprotein substrate/inhibitor and itraconazole a potent inhibitor of this protein [51,59]. P-gp has a very wide substrate spectrum including antineoplastic drugs, macrolides and calcineurin inhibitors [24,81]. Triazole antifungal agents are also connected with incidences of drug induced liver injury (DILI). A study conducted by Zhou et al. found that itraconazole was most often connected with liver injury, followed by voriconazole and fluconazole [82]. It is assumed that drugs metabolized by CYP2C9, CYP1A2 and CYP3A4 are more likely to cause hepatotoxicity than drugs that are not metabolized by those enzymes. It was shown that azole antifungal drugs inhibit the bile salt export pump in the liver and the multidrug resistance gene product 3. This represents a dual mechanism of liver damage in susceptible patients, However, the exact reason remains unclear [83]. Interestingly, in a newer study, echinocandins were found to be more often associated with DILI than triazole antifungal drugs, with higher mortality rates. The authors, however, explain that this result might be biased because echinocandins are often used in patients with liver damage themselves, and underline the need for further studies on that topic [82]. In the context of triazole DDIs, the possibility of QT interval prolongation must also be kept in mind. Fluconazole, itraconazole, voriconazole, and posaconazole are connected with prolongation of the QT interval themselves in different mechanisms [84]. In hematologic patients, special attention should be paid to voriconazole [85]. This is in line with the cases of QT prolongation in pediatric patients reported in EudraVigilance ^®^ in the timespan from 2017 to 2022. The greatest incidence was observed for voriconazole (12 reported cases), followed by fluconazole (4 reported cases) and posaconazole (2 reported cases). For itraconazole, no case was reported. In that context, the risk factors and drug interaction related factors increasing the risk for QT prolongation have to be kept in mind. Uncorrected electrolyte disturbances (also drug-induced electrolyte disturbances), relevant genetic disorders that are usually omitted (e.g., congenital long QT), using two or more drugs prolonging the QT interval and using drugs reducing the clearance of those prolonging the QT interval are of great importance in pediatric cancer patient management [86]. On the other hand, triazole antifungal drugs, similarly to liposomal amphotericin B, have a wide spectrum of antifungal activity including *Coccidioides immitis*, *Mucorales* (posaconazole and isavuconazole), *Fusarium* spp. (voriconazole, posaconazole, isavuconazole and to a lesser extent itraconazole), *Scedosporium* spp. (itraconazole, voriconazole, posaconazole, and isavuconazole), *Blastomyces dermatitidis* and *Histoplasma capsulatum* (itraconazole, voriconazole, posaconazole, isavuconazole and to a lesser extent fluconazole) [67]. Their molecular properties allow them to penetrate to the central nervous system, and achieve up to 100% of serum concentration in the cerebrospinal fluid and brain tissue [72]. What is important is that the triazole antifungal agents are approved not only for the treatment, but also for prophylaxis in the pediatric population. Fluconazole may be used even in neonates, while voriconazole and posaconazole may be used from the age of 2; itraconazole and isavuconazole are only approved for the adult population, and may be used in children if the clinical benefits overweight the risk factors [40,55,57,87,88]. Triazole agents are available in oral formulations, facilitating their administration and preventing the injection site adverse drug reactions. However, gastric acidity may influence pharmacokinetic properties of orally administered drugs, like it was shown for posaconazole [67].

## 4. Conclusions

In conclusion, use of antifungal drugs is inevitable in critically ill patients, which is also reflected in current recommendations. Children with hemato-oncology malignances are particularly vulnerable to drug-drug interactions due to complicated dosing regiments and the need for concomitant use of numerous medications.

This review was an attempt to characterize potential DDIs between antifungal agents and commonly used pharmaceutics in European pediatric hemato-oncology settings. Special attention was paid to the treatment of acute lymphoblastic leukemia and detection of drugs with most the favorable DDI profile.

Triazoles require attention because, due to the inhibition of enzymes involved in the metabolism of numerous drugs they are often responsible for DDIs. This is also reflected in the numbers of spontaneous ADR reports obtained from countries of the European Union.

Although lipid/liposomal formulations of amphotericin B are in use for 30 years now, they are still a cornerstone in the treatment of numerous fungal infections and also, because of a very limited number of potential DDIs in the pediatric hemato-oncology settings. Perhaps, this is also the reason why there are scientific attempts to introduce L-AmB into the prophylaxis of IFD in children with blood cancers.

Echinocandins also present a favorable potential DDI profile, However, taking into consideration the relatively narrow activity spectrum, their clinical use may be limited.

There are numerous available drug interaction checking tools, that might facilitate the identification of potential DDIs, however, they might also lead to alert fatigue. Our review showed that it is important not to stick just to one tool, as the results may differ between the particular checking programs.

Causes for those alterations result from no standardized definition of the severity of DDIs in the databases, limitations in data collection of adverse drug interactions, and lack of standardized methods to evaluate the clinical relevance of detected potential interactions. The alerts are mostly based on limited data from single case reports.

Therefore, in each clinical situation, especially in the case of insufficient data or discrepancy between databases, careful examination of a patient’s medication list by an experienced clinical pharmacist or clinical pharmacologist is recommended. Furthermore, the particular patient situation has to be taken into consideration, before clinical decision-making. It is recommended to introduce a multidisciplinary advisory team, including clinical pharmacists, clinical pharmacologists and microbiologists, to ensure and support proper pharmacotherapy decision-making in the individual patient. Also, systemic approaches like antifungal stewardships implementation, may be a promising approach for treatment optimization.

## Figures and Tables

**Table 1:** Potential DDI between antifungal agents and therapeutics used in pediatric ALL treatment.

Concomitantly Used Drug	L-AmB	FCZ		VCZ		PCZ		ICZ		ISZ		MYC	CASP	
Prednisolone	D	D		D		D		D		D				
Prednisone	D	D, M		D		D		D		D				
Dexamethasone	D	D		D	M	D	M	D	M	D			M	D
Hydrocortisone	D	D		D		D		D		D				
Methotrexate	D	D		D		D		D					D	
6-mercaptopurine														
Thioguanine		D		D		D		D					D	
Cytarabine														
Fludarabine														
Vindeisine														
Vincristine		D	M	D, M		D, M		D, M		D				
Doxorubicin		D	M	D	M	D	M	D	M	D				
Daunorubicin		D		D,		D		D		D				
Cyclophosphamide	D	M								D				
Ifosfamide	D	M		M		M				D				
Etoposide		D		D		D		D		D				
PEG-l-asparaginase		D		D		D		D					D	
Erwinia asparaginase		D		D		D		D					D	
Filgrastim														
Blinatumomab														
Bortezomib				D		D		D						
Imatinib		D		D		D		D		D				
Ruxolitinib		D, M		D, M		D, M		D, M						
Nelarabine														
Inotuzumab ozogamicin		D	M	D	M	D	M	M						
Tisagenlecleucel														
Ciprofloxacin		D	M	D	M	D	M	D, M		D				
Clarithromycin		M		D	M	M		D	M	D	M			
Roxithromycin														
Moxifloxacin		D		D, M		D, M		M						
Clindamycin				D				D						
Erythromycin		D	M	D, M		D	M	D		D				
Piperacillin/tazobactam														
Ceftazidime	D													
Cefepime														
Meropenem														
Imipenem														
Cilastatin														
Amikacin	D													
Tobramycin	D													
Gentamycin	D													
Vancomycin	D													
Teicoplanin														
Colistin	D													
Metronidazole		D	M	D	M	D	M	M						
Cotrimoxazole		M												
Dapsone								D						
Atovaquone														
Acyclovir														
Valganciclovir	D													
Foscarnet	D	D	M	D	M	D	M	M						
Famotidine		D		D		D		D						
Pantoprazole	D	D		D, M		D		D						
Omeprazole	D	D, M		D	M	D, M		D						
Lansoprazole	D	D		D	M	D		D						
Dexlansoprazole	D	D		D		D, M		D						
Rabeprazole	D	D		D, M		D		D						
Esomeprazole	D	D, M		D, M		D	M	D						
Ondansetron		D	M	D	M	D	M	M		D				
Tropisetron														
Granisetron		D	M	D	M	D	M	D		D				
Metoclopramide														
Aprepitant		D	M	D	M	D	M	D	M	D				
Dimenhydrinate														
Lactulose		D		D		D								
Polyethylene glycol		D		D		D								
Sodium picosulphate														
Bisacodyl		D		D		D								
Phenytoin		D	M	M		D	M	D		D	M		M	D
Phenobarbital				D		D		D		D			D	
Carbamazepine		D	M	D	M	D, M		D	M	D	M		M	
Gabapentin														
Levetiracetam														
Enoxaparin														
Dalteparin														
Allopurinol														
Rasburicase														
Drotaverine														
Papaverine		D, M		D		D, M								
Morphine						D,		D, M		D				
Tramadol		D	M	D	M	D	M	D		D				
Oxycodone		D, M		D, M		D, M		D, M		D				
Fentanyl		D, M		D, M		D, M		D, M		D				
Buprenorphine		D	M	D	M	D	M	D	M	D				
Methadone		D	M	D, M		D, M		D	M	D				
Nalbuphine														
Acetaminophen/paracetamol														
Moderate				Major						Contraindicated				

D—Drugs.com^®^, M—Micromedex^®^. Drugs.com^®^ database: Major—highly clinically significant; avoid combinations, the risk of the interaction outweighs the benefit. Moderate—moderately clinically significant; usually avoid combinations, use it only under special circumstances. Micromedex^®^ database: Contraindicated—the drugs are contraindicated for concurrent use. Major—the interaction may be life-threatening and/or require medical intervention to minimize or prevent serious adverse effects. Moderate—the interaction may result in exacerbation of the patient’s condition and/or require an alteration in therapy. L-AmB—liposomal amphotericin B, FCZ—fluconazole, VCZ—voriconazole, PCZ—posaconazole, ICZ—itraconazole, ISZ—izavuconazole, MYC—micafungin, CASP—caspofungin.

**Table 2:** DDI’s share in the total number of ADR notifications in the pediatric population from 2017 to 2022 based on EudraVigilance^®^ reports.

Year	Amphotericin B Formulations/ADR Number for L-AmB N of DDI (Total Serious ADR Number)	%	FCZ N of DDI (Total Serious ADR Number)	%	VCZ N of DDI (Total Serious ADR Number)	%	PCZ N of DDI (Total Serious ADR Number)	%	ICZ N of DDI (Total Serious ADR Number)	%	ISZ N of DDI (Total Serious ADR Number)	%	MYC N of DDI (Total Serious ADR Number)	%	CASP N of DDI (Total Serious ADR Number)	%
2017	3/2 (126)	2.3/1.5	7 (67)	10.4	19 (144)	13.1	5 (31)	16.1	9 (29)	31.0	0 (5)	0.0	1 (29)	3.4	2 (23)	8.7
2018	3/0 (135)	2.2/0.0	7 (48)	14.6	13 (86)	15.1	4 (24)	16.7	2 (8)	25.0	0 (3)	0.0	0 (20)	0.0	1 (26)	3.8
2019	0/0 (117)	0.0/0.0	4 (46)	8.7	8 (109)	7.3	4 (19)	21.0	2 (13)	15.3	1 (9)	11.1	0 (22)	0.0	1 (26)	3.8
2020	1/1 (97)	1.0/1.0	2 (32)	6.2	7 (72)	9.7	3 (32)	9.3	1 (10)	10.0	0 (12)	0.0	0 (13)	0.0	2 (39)	5.1
2021	2/1 (121)	1.6/0.8	4 (63)	6.3	12 (97)	12.4	7 (34)	20.6	0 (20)	0.0	1 (11)	9.1	0 (21)	0.0	5 (35)	14.2
2022	2/0 (97)	2.1/0.0	7 (61)	11.5	8 (89)	9.0	4 (35)	11.4	2 (6)	33.3	0 (6)	0.0	0 (15)	0.0	3 (29)	10.3

L-AmB—liposomal amphotericin B, FCZ—fluconazole, VCZ—voriconazole, PCZ—posaconazole, ICZ—itraconazole, ISZ—izavuconazole, MYC—micafungin, CASP—caspofungin, DDI—drug-drug interaction, ADR—adverse drug reaction.

## Data Availability

Not applicable.

## References

[1] Park H.J., Moon E.K., Yoon J.Y., Oh C.M., Jung K.W., Park B.K., Shin H.Y., Won Y.J. (2016). Incidence and survival of childhood cancer in Korea. Cancer Res. Treat..

[2] Linabery A.M., Ross J.A. (2008). Trends in childhood cancer incidence in the U.S. (1992–2004). Cancer.

[3] Bao P., Zheng Y., Wang C., Gu K., Jin F., Lu W. (2009). Time Trends and Characteristics of Childhood Cancer Among Children Age 0–14 in Shanghai. Pediatr. Blood Cancer.

[4] Desandes E., Clavel J., Berger C., Bernard J.L., Blouin P., De Lumley L., Demeocq F., Freycon F., Gembara P., Goubin A. (2004). Cancer incidence among children in France, 1990–1999. Pediatr. Blood Cancer.

[5] Stack M., Walsh P.M., Comber H., Ryan C.A., O’Lorcain P. (2007). Childhood cancer in Ireland: A population-based study. Arch. Dis. Child..

[6] Dreifaldt A.C., Carlberg M., Hardell L. (2004). Increasing incidence rates of childhood malignant diseases in Sweden during the period 1960–1998. Eur. J. Cancer.

[7] Mejía-Aranguré J.M., Bonilla M., Lorenzana R., Juárez-Ocaña S., de Reyes G., Pérez-Saldivar M.L., González-Miranda G., Bernáldez-Ríos R., Ortiz-Fernández A., Ortega-Alvarez M. (2005). Incidence of leukemias in children from El Salvador and Mexico City between 1996 and 2000: Population-based data. BMC Cancer.

[8] Fajardo-Gutiérrez A., Juárez-Ocaña S., González-Miranda G., Palma-Padilla V., Carreón-Cruz R., Ortega-Alvárez M.C., Mejía-Arangure J.M. (2007). Incidence of cancer in children residing in ten jurisdictions of the Mexican Republic: Importance of the Cancer registry (a population-based study). BMC Cancer.

[9] Inaba H., Greaves M., Mullighan C.G. (2013). Acute lymphoblastic leukaemia. Lancet.

[10] Rizzari C., Lanvers-Kaminsky C., Valsecchi M.G., Ballerini A., Matteo C., Gerss J., Wuerthwein G., Silvestri D., Colombini A., Conter V. (2019). Asparagine levels in the cerebrospinal fluid of children with acute lymphoblastic leukemia treated with pegylated-asparaginase in the induction phase of the AIEOP-BFM ALL 2009 study. Haematologica.

[11] Koka A., Saygin C., Uzunaslan D., Ozdemir N., Apak H., Celkan T. (2014). A 17-year experience with ALL-BFM protocol in acute lymphoblastic leukemia: Prognostic predictors and interruptions during protocol. Leuk. Res..

[12] Khan S., Anwar S., Latif M.F., Farooq A., Faizan M. (2020). Induction-remission response in peadiatric acute lymphoblastic leukaemia, Lahore protocol versus UKALL 2011 interim guidelines. J. Pak. Med. Assoc..

[13] Donadieu J., Auclerc M.F., Baruchel A., Perel Y., Bordigoni P., Landman-Parker J., Leblanc T., Cornu G., Sommelet D., Leverger G. (2000). Prognostic study of continuous variables (white blood cell count, peripheral blast cell count, haemoglobin level, platelet count and age) in childhood acute lymphoblastic leukaemia. Analysis of a population of 1545 children treated by the french acute lym. Br. J. Cancer.

[14] Conter V., Bartram C.R., Valsecchi M.G., Schrauder A., Panzer-Grümayer R., Möricke A., Aricò M., Zimmermann M., Mann G., De Rossi G. (2010). Molecular response to treatment redefines all prognostic factors in children and adolescents with B-cell precursor acute lymphoblastic leukemia: Results in 3184 patients of the AIEOP-BFMALL 2000 study. Blood.

[15] Hunger S.P., Raetz E.A. (2020). How I treat relapsed acute lymphoblastic leukemia in the pediatric population. Blood.

[16] Brown P.A., Ji L., Xu X., Devidas M., Hogan L.E., Borowitz M.J., Raetz E.A., Zugmaier G., Sharon E., Bernhardt M.B. (2021). Effect of Postreinduction Therapy Consolidation With Blinatumomab vs Chemotherapy on Disease-Free Survival in Children, Adolescents, and Young Adults With First Relapse of B-Cell Acute Lymphoblastic Leukemia: A Randomized Clinical Trial. JAMA.

[17] Grigull L., Beier R., Schrauder A., Kirschner P., Loening L., Jack T., Welte K., Sykora K.W., Schrappe M. (2003). Invasive fungal infections are responsible for one-fifth of the infectious deaths in children with ALL. Mycoses.

[18] O’Reilly M.A., Govender D., Kirkwood A.A., Vora A., Samarasinghe S., Khwaja A., Grandage V., Rao A., Ancliff P., Pavasovic V. (2019). The incidence of invasive fungal infections in children, adolescents and young adults with acute lymphoblastic leukaemia/lymphoma treated with the UKALL2011 protocol: A multicentre retrospective study. Br. J. Haematol..

[19] Wang S.S., Kotecha R.S., Bernard A., Blyth C.C., McMullan B.J., Cann M.P., Yeoh D.K., Bartlett A.W., Ryan A.L., Moore A.S. (2019). Invasive fungal infections in children with acute lymphoblastic leukaemia: Results from four Australian centres, 2003–2013. Pediatr. Blood Cancer.

[20] Gründahl M., Wacker B., Einsele H., Heinz W.J. (2020). Invasive fungal diseases in patients with new diagnosed acute lymphoblastic leukaemia. Mycoses.

[21] Klein K., Hasle H., Abrahamsson J., De Moerloose B., Kaspers G.J.L. (2018). Differences in infection prophylaxis measures between paediatric acute myeloid leukaemia study groups within the international Berlin–Frankfürt–Münster (I-BFM) study group. Br. J. Haematol..

[22] Groll A.H., Pana D., Lanternier F., Mesini A., Ammann R.A., Averbuch D., Castagnola E., Cesaro S., Engelhard D., Garcia-Vidal C. (2021). 8th European Conference on Infections in Leukaemia: 2020 guidelines for the diagnosis, prevention, and treatment of invasive fungal diseases in paediatric patients with cancer or post-haematopoietic cell transplantation. Lancet Oncol..

[23] Lehrnbecher T., Fisher B.T., Phillips B., Beauchemin M., Carlesse F., Castagnola E., Duong N., Dupuis L.L., Fioravantti V., Groll A.H. (2020). Clinical practice guideline for systemic antifungal prophylaxis in pediatric patients with cancer and hematopoietic stem-cell transplantation recipients. J. Clin. Oncol..

[24] Chau M.M., Daveson K., Alffenaar J.W.C., Gwee A., Ho S.A., Marriott D.J.E., Trubiano J.A., Zhao J., Roberts J.A., Chang C.C. (2021). Consensus guidelines for optimising antifungal drug delivery and monitoring to avoid toxicity and improve outcomes in patients with haematological malignancy and haemopoietic stem cell transplant recipients. Intern. Med. J..

[25] El Rouby N., Lima J.J., Johnson J.A. (2018). Proton pump inhibitors: From CYP2C19 pharmacogenetics to precision medicine. Expert Opin. Drug Metab. Toxicol..

[26] Hussain M.I., Reynolds T.L., Zheng K. (2019). Medication safety alert fatigue may be reduced via interaction design and clinical role tailoring: A systematic review. J. Am. Med. Inform. Assoc..

[27] Gonzalez D., Sinha J. (2022). Pediatric Drug-Drug Interaction Evaluation: Drug, Patient Population, and Methodological Considerations. J. Clin. Pharmacol..

[28] Feinstein J., Dai D., Zhong W., Freedman J., Feudtner C. (2015). Potential drug-drug interactions in infant, child, and adolescent patients in Children’s Hospitals. Pediatrics.

[29] Dai D., Feinstein J.A., Morrison W., Zuppa A.F., Feudtner C. (2016). Epidemiology of polypharmacy and potential drug-drug interactions among pediatric patients in ICUs of U.S. children’s hospitals. Pediatr. Crit. Care Med..

[30] Fernández de Palencia Espinosa M.A., Díaz Carrasco M.S., Fuster Soler J.L., Ruíz Merino G., De la Rubia Nieto M.A., Espuny Miró A. (2014). Pharmacoepidemiological study of drug-drug interactions in onco-hematological pediatric patients. Int. J. Clin. Pharm..

[31] De Palencia Espinosa M.Á.F., Carrasco M.S.D., Salinas A.S., De La Rubia Nieto A., Miró A.E. (2017). Potential drug-drug interactions in hospitalised haematological patients. J. Oncol. Pharm. Pract..

[32] Balk T.E., van der Sijs I.H., van Gelder T., Janssen J.J.B., van der Sluis I.M., van Leeuwen R.W.F., Engels F.K. (2017). Drug-drug interactions in pediatric oncology patients. Pediatr. Blood Cancer.

[33] Cesaro S., Milano G.M., Aversa F. (2011). Retrospective survey on the off-label use of posaconazole in pediatric hematology patients. Eur. J. Clin. Microbiol. Infect. Dis..

[34] Vicenzi E.B., Calore E., Decembrino N., Berger M., Perruccio K., Carraro F., Rossin S., Putti M.C., Molinaro M., Tridello G. (2018). Posaconazole oral dose and plasma levels in pediatric hematology-oncology patients. Eur. J. Haematol..

[35] Kheshti R., Aalipour M., Namazi S. (2016). A comparison of five common drug-drug interaction software programs regarding accuracy and comprehensiveness. J. Res. Pharm. Pract..

[36] Holoxan Product Information. https://www.medicines.org.uk/emc/product/1834/smpc#gref.

[37] Moriyama B., Henning S.A., Leung J., Falade-Nwulia O., Jarosinski P., Penzak S.R., Walsh T.J. (2012). Adverse interactions between antifungal azoles and vincristine: Review and analysis of cases. Mycoses.

[38] van Schie R.M., Brüggemann R.J.M., Hoogerbrugge P.M., te Loo D.M.W.M. (2011). Effect of azole antifungal therapy on vincristine toxicity in childhood acute lymphoblastic leukaemia. J. Antimicrob. Chemother..

[39] Harnicar S., Adel N., Jurcic J. (2009). Modification of vincristine dosing during concomitant azole therapy in adult acute lymphoblastic leukemia patients. J. Oncol. Pharm. Pract..

[40] Cresemba Product Information. https://www.ema.europa.eu/en/documents/product-information/cresemba-epar-product-information_pl.pdf.

[41] Gleevec Product Information. https://www.ema.europa.eu/en/medicines/human/EPAR/glivec#product-information-section.

[42] Velcade Product Information. https://www.ema.europa.eu/en/documents/product-information/velcade-epar-product-information_en.pdf.

[43] Jakavi Product Information. https://www.ema.europa.eu/en/documents/product-information/jakavi-epar-product-information_en.pdf.

[44] Lato M.W., Przysucha A., Grosman S., Zawitkowska J., Lejman M. (2021). The new therapeutic strategies in pediatric t-cell acute lymphoblastic leukemia. Int. J. Mol. Sci..

[45] Besponsa Product Information. https://www.ema.europa.eu/en/documents/product-information/besponsa-epar-product-information_en.pdf.

[46] Yu Z., Liao X. (2022). Torsade de Pointes/QT Prolongation Associated with Antifungal Triazoles: A Pharmacovigilance Study Based on the U.S. FDA Adverse Event Reporting System (FAERS). J. Pharm. Pharm. Sci..

[47] Pasternak Y., Shechter N., Loebstein R., Markovits N., Gueta I., Halkin H., Yarden-Bilavsky H. (2019). Voriconazole-induced QTc prolongation in a paediatric population. Acta Paediatr. Int. J. Paediatr..

[48] Kebriaei P., Cutler C., De Lima M., Giralt S., Lee S.J., Marks D., Merchant A., Stock W., Van Besien K., Stelljes M. (2018). Management of important adverse events associated with inotuzumab ozogamicin: Expert panel review. Bone Marrow Transplant..

[49] Hibma J.E., Kantarjian H.M., DeAngelo D.J., Boni J.P. (2019). Effect of inotuzumab ozogamicin on the QT interval in patients with haematologic malignancies using QTc-concentration modelling. Br. J. Clin. Pharmacol..

[50] Munir F., He J., Connors J., Garcia M., Gibson A., McCall D., Nunez C., Dinh C.N., Robusto L., Roth M. (2023). Translational advances in the treatment of childhood acute lymphoblastic leukemia: Narrative review of current and emerging molecular and immunotherapies. Transl. Pediatr..

[51] Heiskanen T., Backman J.T., Neuvonen M., Kontinen V.K., Neuvonen P.J., Kalso E. (2008). Itraconazole, a potent inhibitor of P-glycoprotein, moderately increases plasma concentrations of oral morphine. Acta Anaesthesiol. Scand..

[52] Kharasch E.D., Hoffer C., Whittington D., Sheffels P. (2003). Role of P-glycoprotein in the intestinal absorption and clinical effects of morphine. Clin. Pharmacol. Ther..

[53] Flockhart Table P450 Drug Interactions. Indiana University. https://drug-interactions.medicine.iu.edu/MainTable.aspx.

[54] Sancuso Product Information. https://www.ema.europa.eu/en/documents/product-information/sancuso-epar-product-information_en.pdf.

[55] Noxafil Product Information. https://www.ema.europa.eu/en/documents/product-information/noxafil-epar-product-information_en.pdf.

[56] Bunondol Product Information. https://rejestrymedyczne.ezdrowie.gov.pl/api/rpl/medicinal-products/1196/characteristic.

[57] Diflucan. Product Information. https://www.pfizerpro.com.pl/sites/default/files/diflucan_lld_approved_10.12.2020.pdf.

[58] Moody D.E., Liu F., Fang W.B. (2015). Azole antifungal inhibition of buprenorphine, methadone and oxycodone in vitro metabolism. J. Anal. Toxicol..

[59] Fihlman M., Hemmilä T., Hagelberg N.M., Kuusniemi K., Backman J.T., Laitila J., Laine K., Neuvonen P.J., Olkkola K.T., Saari T.I. (2016). Voriconazole more likely than posaconazole increases plasma exposure to sublingual buprenorphine causing a risk of a clinically important interaction. Eur. J. Clin. Pharmacol..

[60] Trimesolphar Product Information. https://rejestrymedyczne.ezdrowie.gov.pl/api/rpl/medicinal-products/1081/characteristic.

[61] Hadjibabaie M., Badri S., Ataei S., Moslehi A.H., Karimzadeh I., Ghavamzadeh A. (2013). Potential drug-drug interactions at a referral hematology-oncology ward in Iran: A cross-sectional study. Cancer Chemother. Pharmacol..

[62] Mitra A.K., Thummel K.E., Kalhorn T.F., Kharasch E.D., Unadkat J.D., Slattery J.T. (1996). Inhibition of sulfamethoxazole hydroxylamine formation by fluconazole in human liver microsomes and healthy volunteers. Clin. Pharmacol. Ther..

[63] Dittrich A.T.M., Draaisma J.M.T., van Puijenbroek E.P., Loo D.M.W.M.t. (2020). Analysis of Reporting Adverse Drug Reactions in Paediatric Patients in a University Hospital in the Netherlands. Pediatr. Drugs.

[64] Pérez García M., Figueras A. (2011). The lack of knowledge about the voluntary reporting system of adverse drug reactions as a major cause of underreporting: Direct survey among health professionals. Pharmacoepidemiol. Drug Saf..

[65] Fabiano V., Mameli C., Zuccotti G.V. (2012). Adverse drug reactions in newborns, infants and toddlers: Pediatric pharmacovigilance between present and future. Expert Opin. Drug Saf..

[66] Chang C.C., Slavin M.A., Chen S.C.A. (2017). New developments and directions in the clinical application of the echinocandins. Arch. Toxicol..

[67] Nett J.E., Andes D.R. (2016). Antifungal Agents: Spectrum of Activity, Pharmacology, and Clinical Indications. Infect. Dis. Clin. N. Am..

[68] Hoffman J.A., Walsh T.J. (2011). Echinocandins in children. Pediatr. Infect. Dis. J..

[69] Cancidas Product Information. https://www.ema.europa.eu/en/documents/product-information/cancidas-epar-product-information_pl.pdf.

[70] Schneeweiss S., Carver P.L., Datta K., Galar A., Johnson M.D., Letourneau A.R., Marty F.M., Nagel J., Najdzinowicz M., Saul M. (2020). Long-term risk of hepatocellular carcinoma mortality in 23220 hospitalized patients treated with micafungin or other parenteral antifungals. J. Antimicrob. Chemother..

[71] Mycamine Product Information. https://ec.europa.eu/health/documents/community-register/2018/20180726141675/anx_141675_pl.pdf.

[72] Ashley E.D. (2019). Antifungal drugs: Special problems treating central nervous system infections. J. Fungi.

[73] FDA Drug Safety Communication: Low Magnesium Levels Can Be Associated with Long-Term Use of Proton Pump Inhibitor Drugs (PPIs). https://www.fda.gov/drugs/drug-safety-and-availability/fda-drug-safety-communication-low-magnesium-levels-can-be-associated-long-term-use-proton-pump.

[74] Kyriakidis I., Tragiannidis A., Munchen S., Groll A.H. (2017). Clinical hepatotoxicity associated with antifungal agents. Expert Opin. Drug Saf..

[75] AmBisome Product Information. https://rejestrymedyczne.ezdrowie.gov.pl/api/rpl/medicinal-products/31/characteristic.

[76] Roman E., Osunkwo I., Militano O., Cooney E., van de Ven C., Cairo M.S. (2008). Liposomal amphotericin B prophylaxis of invasive mold infections in children post allogeneic stem cell transplantation. Pediatr. Blood Cancer.

[77] Mehta P., Vinks A., Filipovich A., Vaughn G., Fearing D., Sper C., Davies S. (2006). High-dose weekly amBisome antifungal prophylaxis in pediatric patients undergoing hematopoietic stem cell transplantation: A pharmacokinetic study. Biol. Blood Marrow Transplant..

[78] Groll A.H., Rijnders B.J.A., Walsh T.J., Adler-Moore J., Lewis R.E., Brüggemann R.J.M. (2019). Clinical Pharmacokinetics, Pharmacodynamics, Safety and Efficacy of Liposomal Amphotericin B. Clin. Infect. Dis..

[79] Tornio A., Filppula A.M., Niemi M., Backman J.T. (2019). Clinical Studies on Drug-drug Interactions Involving Metabolism and Transport: Methodology, Pitfalls, and Interpretation. Clin. Pharmacol. Ther..

[80] Wang N.N., Wang X.G., Xiong G.L., Yang Z.Y., Lu A.P., Chen X., Liu S., Hou T.J., Cao D.S. (2022). Machine learning to predict metabolic drug interactions related to cytochrome P450 isozymes. J. Cheminform..

[81] Finch A., Pillans P. (2014). P-glycoprotein and its role in drug-drug interactions. Aust. Prescr..

[82] Zhou Z.-X., Yin X.-D., Zhang Y., Shao Q.-H., Mao X.-Y., Hu W.-J., Shen Y.-L., Zhao B., Li Z.-L. (2022). Antifungal Drugs and Drug-Induced Liver Injury: A Real-World Study Leveraging the FDA Adverse Event Reporting System Database. Front. Pharmacol..

[83] Andrade R.J., Chalasani N., Björnsson E.S., Suzuki A., Kullak-Ublick G.A., Watkins P.B., Devarbhavi H., Merz M., Lucena M.I., Kaplowitz N. (2019). Drug-induced liver injury. Nat. Rev. Dis. Prim..

[84] Chai S., Zhan J.-L., Zhao L.-M., Liu X.-D. (2022). Safety of triazole antifungals: A pharmacovigilance study from 2004 to 2021 based on FAERS. Ther. Adv. Drug Saf..

[85] Gueta I., Loebstein R., Markovits N., Kamari Y., Halkin H., Livni G., Yarden-Bilavsky H. (2017). Voriconazole-induced QT prolongation among hemato-oncologic patients: Clinical characteristics and risk factors. Eur. J. Clin. Pharmacol..

[86] Khatib R., Sabir F.R.N., Omari C., Pepper C., Tayebjee M.H. (2021). Managing drug-induced QT prolongation in clinical practice. Postgrad. Med. J..

[87] Vfend Product Information. https://www.ema.europa.eu/en/documents/product-information/vfend-epar-product-information_en.pdf.

[88] Orungal Product Information. https://rejestrymedyczne.ezdrowie.gov.pl/rpl/search/public.

